# Spectrum of HRCT Scan Chest Findings in COVID-19 Patients as Categorized by Modified CO-RADS Classification

**DOI:** 10.12669/pjms.38.4.4687

**Published:** 2022

**Authors:** Sumera Tabassum, Shahbaz Haider, Shaista Shaukat

**Affiliations:** 1 Dr. Sumera Tabassum, Assistant Professor, Department of Radiology, Jinnah Postgraduate Medical Center, Karachi, Pakistan; 2 Prof. Shahbaz Haider, Medical Unit-I, Jinnah Postgraduate Medical Center, Karachi, Pakistan; 3 Dr. Shaista Shaukat, Associate Professor, Department of Radiology, Jinnah Postgraduate Medical Center, Karachi, Pakistan

**Keywords:** Modified CO-RADS classification, Covid-19

## Abstract

**Objectives:**

The Dutch Radiological Society developed CO-RADS classification, a system for the classification of CT scan chest findings among suspected COVID-19 patients. However due to some important issues it was modified by authors and then applied on our study population. The objective was to study the spectrum of lungs involvement as concluded by HRCT scan chest finding and classifying it using the “Modified CO-RADS classification”

**Methods:**

This cross-sectional study was conducted jointly by the departments of Medicine and Radiology, JPMC from January 16, 2021 to April 30, 2021. This study includes suspected cases of COVID-19 patients aged between 18-80 years who came for HRCT chest. Their data variables were recorded. HRCT findings were classified using “Modified CO-RADS classification”. Patients’ results of real time PCR for COVID-19 were also followed.

**Results:**

A total of 78 patients presented to the study department during this study period. Of them 85.8% were male (n=67) and 14.2% were female (n=11). Out of the 78 patients, 58 were tested positive for COVID-19 on first RT-PCR on follow up. Among positive two patients (3.4%) had CO-RADS-1, 4 patients (7%) had CO-RADS-2, 19 patients (32.75%) had CO-RADS-3, 21 patients (36.2%) had CO-RADS-4 while 12 patients (20.7%) had CO-RADS-5 category. (CO-RAD-6 category was omitted). Of the patient who had negative results on RT-PCR, five patients had CO-RADS-4 while three patients had CO-RADS-5. On repeat RT-PCR all (8/8) patients of category IV and V proved Covid-19 positive.

**Conclusion:**

HRCT scan chest can be used for quicker diagnosis of COVID-19 patients in patients with respiratory complaints in whom prompt diagnosis is required and when RT-PCR investigation process would be taking prolonged time due to over burden during pandemic situation. “CO-RADS classification after modification” proved more effective communicative tool to label and understand the severity of lung involvement in Covid-19 disease.

## INTRODUCTION

Coronavirus is a virus of the family of viruses named orthocoronavirinae which led to a pandemic commencing by the end of 2019 and hence named COVID-19. This pandemic started in the city of Wuhan, China and later involved all parts of the world. The mortality of COVID-19 is reported as 5.6% in China and 15.2% in other parts of the world.^1^ The diagnostic test available for the diagnosis of COVID-19 virus is RT-PCR (Real Time Polymerase chain reaction ) with a sensitivity of 42% to 83%. Certain factors affect the accuracy of the test amongst which are viral load of patient, adequate sample collection.^2^ The single real time PCR test used for COVID-19 may not be positive in all real Covid-19 patients as may be explainable from its given sensitivity of 42 to 83%, however being convenient and easily performable, it has been mainstay in diagnosis and management. But in pandemic when test was widely needed for all level of suspected patients having fever, mild to severe respiratory symptoms to follow up test in positive cases, its kits supply has been facing shortages from time to time.

CO-RADS, Coronavirus Reporting and data system, is a computed tomography-based classification assessing the radiologic manifestations of COVID-19. It was developed by the Dutch Radiological society. It can be used in correlation with clinical clues to help reach the diagnosis and commence management.^3^ However, as this classification has been adapted from BIRAD classification for carcinoma breast by Dutch Radiological society,^3^ oversimplification is seen in its adaptation. In BIRAD MRI imaging done is of same area i.e., breast, to assess signal changes produced by the pathology due to malignancy in same imaged area i.e. breast. In Covid-19 infection simple viral infection positivity concluded from Positive Real Time-PCR from nasopharyngeal swab should not be equalized with positive Biopsy result for malignancy as is considered and classified in BIRAD classification. For this important difference in area under study and pathogenesis, CO-RADS classification is used in this study after discussions and then modification by all three authors and name given is “Modified CO-RADS classification” and described in Methods. Justifications and benefits would be discussed in discussion section of article.

Real Time PCR results can be negative in some patients or tests being delayed on reasons mentioned, in patients having clinical features suspected of COVID-19. In such instances, CT scan chest findings can help in the diagnosis. The foremost important step in the management of COVID-19 patients is isolation and prevention of further spread of this deadly virus among contacts. Emergent CT scan findings can help initiate important steps in the management of COVID-19 patients. Our objective was to “study the spectrum of lungs involvement as concluded by HRCT scan chest finding and classifying it using the “Modified CO-RADS classification.”

## METHODS

This is a cross-sectional study conducted by the Department of Radiology and Medicine, Jinnah Postgraduate Medical Center, Karachi. The study was approved by the Ethical Review Board of Jinnah Postgraduate Medical Center (Ref: NO.F.2-81.2021-GENL/56950/JPMC, Dated March 26, 2021). Study was done from January 16, 2021 to April 30, 2021. Subjects were selected from patients sent by physicians to Radiology Department on suspicion of Covid-19. Age range selected was 16-80 years. Other Inclusion criteria included: Patients with cough and\or breathlessness with fever or without fever or with anosmia. Exclusion criteria included history of lung Surgery , Pulmonary Tuberculosis or lung malignancy. Informed consent was taken from all patients before including them in the study.

All patients selected were investigated by HRCT Chest. “Modified CO-RADS” classification system was applied. The Modified CO-RADS classification is defined as follows:

### “CO-RADS category 0:

This category is labeled when the scans don’t meet the quality required for classification or due to the presence of artifacts such as breathing abnormalities or cough.”

### “CO-RADS category 1

This category implies an extremely low level of suspicion for the diagnosis of COVID-19. No nodules are found in category 1 of CO-RADS classification. If present they are only due to benign conditions or previously diagnosed conditions such as emphysema, fibrosis, pneumonia, perifissural nodules, or lung tumour.”

### “CO-RADS category 2:

This category implies a low level of suspicion for COVID-19. The CT scan findings are attributable not to covid-19 but to other infectious causes such as bronchitis, bronchiolitis, and some forms of pneumonia.”

### “CO-RADS category 3:

This category is selected when findings are equivocal for Covid-19 lung pathology as these findings are also compatible for other viral pneumonias or non infectious causes. Perihilar ground glass opacities along with pulmonary nodules, extensive ground glass opacities which are homogenous, or with interlobular septal thickening are found. Pleural effusion may or may not be there. It also includes small ground glass opacities which are neither centrilobular nor closed to pleura. It also includes consolidation patterns compatible with organizing pneumonia too and thus not typical of pattern of Covid-19. Thus, this category is suggestive of COVID-19 but can be due to other diseases as well.”

### “CO-RADS category 4:

This category involves high level of suspicion for COVID-19. Imaging usually shows ground glass haze with or without consolidations but not in contact with visceral pleura or present only unilaterally and are present mostly in peribronchovascular distribution. Keeping these differentiating points in mind (not in contact with visceral pleura or only unilaterally and mostly in peribronchovascular distribution) other features of CO-RADS category 5 described below may be present and acceptable for labeling this category.”

### “CO-RADS category 5:

This category carries very high level of suspicion of COVID-19. Imaging usually shows ground glass haze with or without consolidations close to visceral pleura including fissures. Distribution is multifocal as well as bilateral. Vicinity to major or minor fissure is also considered favoring for this category i.e., not limited to periphery when fissures involved. Subpleural sparing may be accepted. Findings as Reverse halo sign, ground glass with extensive subplueral consolidation and air bronchogram may be present.”

### CO-RADS category 6:

Omitted. (This category was said to be “proven COVID-19, as signified by positive PCR test results for Covid 19 virus specific nucleic acid “ on sample taken most likely from nasopharynx as is recommended practice worldwide)

Modification is done by authors and this category six is omitted. As original Dutch CO-RADS classification has been inspired from BI-RAD classification by Dutch Radiological society^3^, oversimplification is seen in its adaptation. In BI-RAD MRI imaging of same area i.e. breast is done to assess signal changes produced by the pathology due to malignancy in same imaged area i.e. breast. In Covid infection simple viral infection positivity concluded from Positive RT-PCR from nasopharyngeal swab should not be equalized with positive biopsy result for malignancy of same area(Breast) as is done in original BI-RADS classification and was adopted for CO-RADS classification. Positive Real Time -PCR in Dutch CO-RADS was taken as equivalent to Positive biopsy in BI-RAD system, which does not look logical and needs review by the experts of field. For this important difference in area under study and pathogenesis differences, CO-RADS classification used in this study is utilized with modification and will be referred to as “Modified CO-RADS classification”.

In this study positive RT-PCR, awaited results of RT-PCR and negative RT-PCR are discussed independently with lung changes as determined by different categories of CO-RADS classification and described above.

Age, demographic variables, and a detailed analysis of symptoms were recorded. Results of RT-PCR of nasopharyngeal swab was planned to be discussed independent of CO-RADS categories. It was recorded in cases when result was available. However, in most cases test was done but result was awaited due to one or other reason. In cases where result was not available or test was not done, advice was given for RT-PCR of COVID-19 and result of all were followed. Patients having negative RT-PCR were advised for second time RT-PCR for Covid-19. Cases where result of first or Second RT-PCR (if advised) could not be acquired, those cases were excluded from study. HRCT scan images were read by a team of three radiologists, two of which were certified radiologists and one radiology resident. The findings were classified using CO-RADS classification. Data was analyzed using SPSS v20.0.

## RESULTS

A total of 78 patients were selected for the study during the study period. Of them 85.8% were males (n=67) and 14.2% were females (n=11). The mean age of our study subjects was 55.03±13.22 years. The minimum age was 23 while the maximum age was 80 years. 19 patients (24.3%) were between ages 20-40, 32 patients (41%) were between age 41-60, 27 patients (34.6%) were between age 61 to 80.

Fifty eight patients were either positive for COVID-19 on RT-PCR at the time of HRCT or awaited result came positive on follow up. It had been discussed that ‘RTA-PCR’ and categories in ‘Modified CO-RAD Classification’ have been considered independent of each other so differing with classical CO-RADS classification (i.e., not placed in Class-6 irrespective of lung findings if RT-PCR was positive as is advised by Dutch CO-RADS classification, rather Class-6 was omitted while modifying Dutch CO-RAD classification as described in Methods).

Among these fifty eight patients, two patients (3.4%) had CO-RADS-1, four patients (7%) had CO-RADS-2, 19 patients (32.75%) had CO-RADS-3, 21 patients (36.2%) had CO-RADS-4 while 12 patients (20.7%) had CO-RADS-5 category.

Of the 20 patient who had negative results on first RT-PCR, seven had CO-RADS-1, two had CO-RADS-2, three had CO-RADS-3, five patients had CO-RADS-4 while 03 patients had CO-RADS-5 category. These patients were advised repeat RT-PCR and on repeat RT-PCR for Covid-19, all patients of category CO-RADS-1 and CO-RADS-2 were again Covid-19 negative, while two of three patients of CO-RADS-3 category came Covid-19 positive (66.6%) and all patients (8/8) of category IV and V came Covid-19 positive on repeat testing.

In our study, the specificity of HRCT in the detection of COVID-19 as per classification’s category IV and V was found to be very high (100%) as supported by first or repeat RT-PCR testing and specificity of diagnosis on basis of CO-RAD IV and V proved more reliable than single RT-PCR test as these negative RT-PCR proved positive on repeat RT-PCR testing. In CO-RADS-3, two of three initially negative on first RT-PCR proved positive on second testing. CO-RADS-1 and CO-RADS-2 came negative again on repeat testing for RT-PCR.

**Fig.1:**
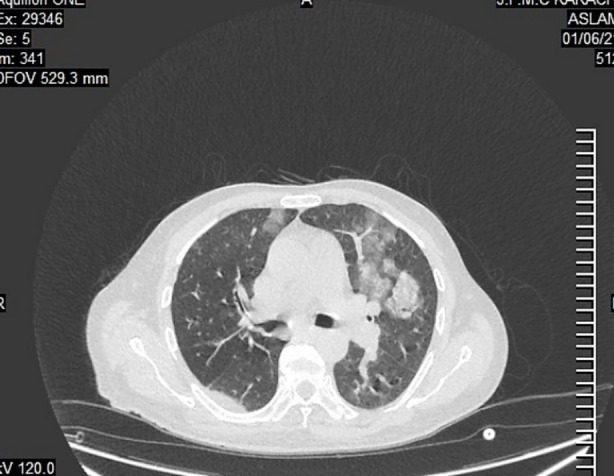
‘CO-RADS Category 3’.

**Fig.2:**
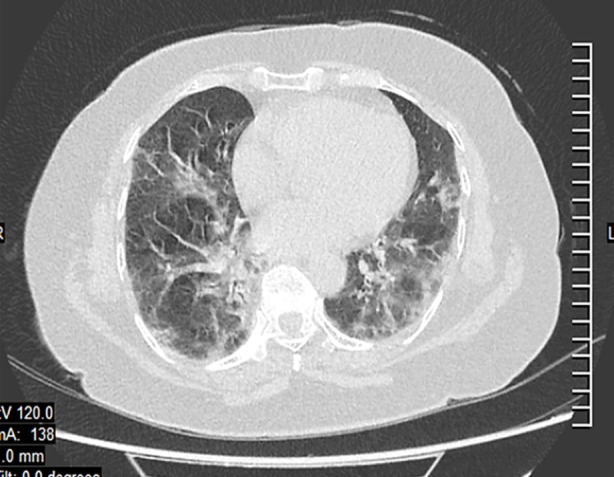
CO-RADS 5 Category.

## DISCUSSION

Lung’s involvement has been major factor in serious morbidity and mortality in Covid-19 affected cases. It has been reported in many studies involving imaging modalities. Asghar MS et al. 4 and Masood L et al.^5^ described lung involvement on X-ray chest. Asghar et al.^4^ reported “bilateral lower zone patchy infiltrates as frequent chest X-ray finding” Regarding modality of CT scan chest Asefi H et al. commented that “at least in earlier stages of the disease, CT may not be valuable as a screening test for COVID-19”.^6^ Most radiologic societies do not recommend Routine screening of Covid-19 by CT chest^7^. Radiological society of Pakistan recommends that when saturation of Oxygen is more than 94%, no chest imaging is recommended in RT-PCR Covid-19 positive patients as it will not add to management.^8^ However in patients with hypoxia or chest infiltrates on X-ray, CT scan modality may prove helpful 8. Sayeed S et al.^9^ and Khaliq M et al.^10^ studied CT scans done in Covid-19 patients, but their focus was to determine the frequency of different degrees of severity of the lung involvement in Covid-19.

Lessman et al. conducted a study to “relate the CT scan findings in patients having suspicion of COVID-19. He used artificial intelligence in predicting the radiologic findings. The study discriminated between positive and negative COVID-19 patients and the area under the curve was reported as 0.95 and 0.88^11^”. Ni et al. performed a similar study. In his study he used a similar model which used deep learning approach to detect CT scan findings among patients with COVID-19. He concluded that “automated methods of detecting CT scan findings can assist clinicians in making diagnosis more effectively using less time and resources”.^12^

Our study shows a high specificity, however, one reason for the high value is the selection of clinically symptomatic patients. Kwee et al. conducted “a meta-analysis on the CO-RADS and RSNA classification system. His findings conclude that CO-RADS one and two do not exclude COVID-19”^13^. In our study two out of nine cases of CO-RADS-1 category were RT-PCR positive and four out of six cases of CO-RADS-2 were RT-PCR positive which also favors the point that CO-RADS-1 and 2 do not exclude COVID-19 infection. It can easily be explained by the fact that CO-RADS categorization is based on lung involvement and its CO-RAD one and two categories may be used as exclusion points for the involvement of lungs in COVID-19 infection, but by no way could be considered to exclude the COVID-19 infection which has a broad spectrum of involvement from asymptomatic, restricted to upper respiratory tract infection, symptomatic for general constitutional symptoms, no lung involvement to severe lung involvement. The sensitivity of Kwee study is similar to our study. He also reports “that the sensitivity of CO-RADS was 90.2%”^13^. Schalekamp et al conducted a study “based on the use of CT scan in emergent conditions for diagnosis of COVID-19”. His study subjects comprised of 1070 patients. He concludes that “CT scan can be effectively used in patients with emergent conditions among patients having symptoms for more than 48 hours to diagnose COVID-19. According to his study results the area under the curve for chest CT was 0.87 when compared with the results for RT-PCR and 0.87 when compared with clinical symptoms”. For patients who need a rapid and reliable diagnosis, chest CT scan is considered an effective modality.^14^

De Smet et al conducted a study to “evaluate the role of chest CT scan in patients with clinically symptomatic and asymptomatic COVID-19 infection. The sensitivity of CO-RADS classification in clinically symptomatic patients was 89% while the specificity was 73% in the same group”. However, the sensitivity of CO-RADS classification in clinically asymptomatic patients was 45% and the specificity was 89%.^15^

Özel et al conducted a study “in a tertiary care setup in Turkey and his study results also concluded that CT scan can be used as an effective modality in evaluating the extent of lung involvement in patients with COVID-19”^16^.

Keeping varying involvement of different organs by COVID-19, and assigning category 6 for Real time PCR positivity for COVID-19, the issue of “category 6” has been arisen repeatedly during the planning of this study. So, it was decided to modify the criteria for reasons discussed below.

“Original CO-RADS criteria” was devised by Dutch Radiological Society on lines of BI-RADS criteria for breast cancer. It was realized by authors of this article that over simplification was done in this process especially adopting ‘category 6’ of BI-RADS. Authors felt very uncomfortable for categorizing patients who, by HRCT Lung were eligible for only category 0 or 1 or 2, but had to be assigned highest category ie. “Category 6” on ‘Real Time PCR positivity alone’ while having minimal or no HRCT findings in favor of Covid -19 lung involvement. Awarding category “CO-RADS 6” merely on PCR positivity looks illogical and it does not correlate with degree of lung involvement. Using original CO-RADS criteria assigning highest category, only due to point that a swab taken from nasopharynx is positive, even after HRCT lungs which shows no or minimal changes with very low or low level of suspicion of lung involvement by COVID-19 (i.e. category 1/2), this highest category (category 6) would be anything else but not the realistic indicator for lung involvement. It is not useful for selecting management strategy for the COVID patients regarding “lung involvement” for which CO-RADS criteria has been mainly devised. Need not to say that lung involvement and lung functions are considered to be most important target organ and most important by function while managing COVID -19 patients by the physicians.

However after omitting category ‘6’ (i.e. after discarding label of ‘category 6’ on ‘Real time PCR positivity’ of nasopharnygeal swab alone for COVID -19) as done by authors in “modified CO-RADS criteria”, its categories have been proved good indicator of severity of lung involvement as anomaly got corrected. It proved good communicative tool among radiologist and physicians with quick understanding of the severity of the disease without need of using additional terminology or phrase for lung involvement esp. in cases of ‘category 6’ (i.e. in Dutch CO-RADS, to communicate real picture one has to say, “category is ‘CO-RADS 6’ but lungs have minimal or no involvement or extensive involvement or one has to say that overall category is 6 but ‘by lung involvement’, it is like 0-4 or even 5 or some other phrase needed for communicating).

It is to be remembered, that patients with lung involvement of CO-RADS category 0/1/2 level but original Dutch criteria forced to label these cases as ‘category 6’ to real time PCR positivity, were bound to be sent for home or general isolation centers after counseling. Such a high grade, category 6 for Nasopharyngeal swab positivity as in original Criteria is expected to do nothing but produce confusions. Thus, category CO-RADS ‘6’ was omitted by discussion of all three authors, i.e. Professor of Medicine, Associate and Assistant Professor of Radiology.

Some Researchers in their studies also tried to avoid this mentioned confusion by excluding the patients who, before the imaging, were known PCR positive for COVID 19 thus eliminating the confusion produced by category 6.^11^,^13^ However, it is not a natural solution of this anomaly. Authors of this article avoided this confusion and treated anomaly for this study by modifying the criteria by totally omitting the category 6.

Furthermore if PCR COVID-19 result could not be arranged earlier due to overburdened kit supply , Co-Rads criteria has been proved a useful diagnostic tool for diagnosis of COVID-19 as higher categories “4 and 5” represents high or very high suspicion for COVID-19 as verified by the studies after getting Positive Real time PCR in follow up after the imaging has been reported as CO-RADS 4 and CO-RADS 5 and thus CO-RADS classification is recommended as helpful tool in deciding management strategy.

### Limitations of the study:

It include a small sample size. Another limiting factor is the duration of study which was a peak for COVID-19 cases. The presence of other lung infections decreases the sensitivity of CT scan.

## CONCLUSION

We can hereby conclude that in addition to assess severity of disease, CT scan can be used for quicker diagnosis of COVID-19 patients in patients with respiratory complaints in whom prompt diagnosis is required and when RT-PCR investigation process would be delaying due to over burden during pandemic situation. “CO-RADS classification after modification” proved flawless effective communicative tool to label and understand the severity of lung involvement in Covid-19 disease and eradicates the chances of labeling overinflated category of CO-RADS 6 when there is “no or low probability” of lung involvement (category 0/1/2) merely due to positive Covid 19 nasopharyngeal swab.

### Authors’ Contributions:

**ST:** Conceived and designed the study, included the patients. evaluated the HRCT scan findings, contributed to drafting and revising of article, contributed to final approval. She is also responsible for the accuracy or integrity of the word.

**SH:** Included the patients, contributed to manuscript writing and critical revising of the article. contributed to critical appraisal of findings with literature and statistical calculations, contributed to final approval of manuscript.

**SS:** Included the patients. evaluated the HRCT scan findings, contributed to drafting and revising of article, contributed to final approval.
